# Ethnic disparities in the relationship between the number of chronic diseases and health status among adults aged ≥45 in Yunnan Province, China

**DOI:** 10.3389/fpubh.2025.1558704

**Published:** 2025-06-10

**Authors:** Rui Deng, Jie Chen, Xinping Wang, Yan Xiao, Ying Chen, Chaofang Yan, Yuan Huang

**Affiliations:** ^1^School of Public Health, Kunming Medical University, Kunming, Yunnan, China; ^2^Yunnan Provincial Key Laboratory of Public Health and Biosafety, Kunming, Yunnan, China; ^3^Yunnan Provincial Key Laboratory of Cross-border Infectious Disease Prevention and New Drug Development, Kunming, Yunnan, China; ^4^Chengdu Seventh People’ s Hospital, Chengdu, Sichuan, China; ^5^Department of Foreign Languages, Kunming Medical University, Kunming, Yunnan, China

**Keywords:** chronic non-communicable diseases, health utility value, activities of daily living, chronic pain, frailty, ethnic minority, *Zhiguo* ethnic minority

## Abstract

**Background:**

Chronic conditions among middle-aged and older adults from ethnic minority backgrounds is becoming increasingly prominent, while there is limited evidence available regarding the influence of NCDs on ethnic minority groups in later life. This study aims to examine the characteristics of NCDs and health status among adults aged ≥45 in multi-ethnic settlement, as well as explore the disparate impacts of chronic condition on ethnically diverse populations.

**Methods:**

This cross-sectional study was conducted in Yunnan Province, China from July to December 2022. Intergroup comparisons were performed using chi-square tests and Kruskal-Wallis H test. Multivariable linear regression and Tobit regression were used to assess the impact of NCDs on health status indicators.

**Results:**

Out of the total 2,710 participants, approximately 57.90% exhibited at least one NCD, with individuals from the *Han* majority exhibiting a higher prevalence (*p* < 0.001). Individuals belong to *Zhiguo* ethnic minority groups had higher mean scores for ADL (15.92, SD = 3.80), chronic pain (2.27, SD = 2.28), and frailty (3.37, SD = 2.61). A significantly negative association was observed between the number of NCDs and the health utility value across all ethnic groups (*p* < 0.001). Conversely, there was a positive relationship between scores for chronic pain and frailty with the number of NCDs for all three ethnic groups respectively; within *Zhiguo* ethnic minority groups alone, there was also a positive correlation between ADL scores and the number of NCDs (*p* < 0.001).

**Conclusion:**

The prevalence of NCDs among adults aged ≥45 in Yunnan province is significantly higher, indicating a substantial burden of NCDs in underdeveloped areas. Although the chronic condition is more prominent among *Han* majority, individuals belonging to *Zhiguo* ethnic minority exhibit poorer health outcomes. And the negative health effects from NCDs to health status are more severe among ethnic minority groups, indicating an urgent need for targeted interventions to address health disparities in multi-ethnic regions.

## 1 Introduction

Non-communicable diseases (NCDs) have emerged as a primary health threat globally for individuals aged 45 and above, resulting in approximately 17 million premature deaths each year (1). Projections indicate that by 2050, NCDs will account for 77.6% of the global disease burden, predominantly affecting low-and middle-income countries (2). These countries bear a disproportionate share of this burden, accounting for 77% of all NCD-related deaths and 86% of premature NCD deaths (1). Furthermore, the increase in mortality and morbidity associated with chronic diseases in low-and middle-income countries surpasses that observed in high-income countries (3). Another significant concern is the age-related rise in the prevalence of chronic diseases, often accompanied by multimorbidity, defined as the presence of multiple chronic conditions simultaneously (4–6).

Chronic conditions have a well-documented negative impact on health, with extensive research highlighting their role in adverse health outcomes over the past decades. Accumulation of chronic conditions has been linked to the onset of frailty (7), cognitive decline (8), mental disorders (9), higher mortality rates (10) and poor quality of life (11). In addition to the observed correlations between NCDs and adverse health outcomes, there exists a significant association between NCDs and socioeconomic characteristics, which further exacerbates health inequities. Individuals with lower socioeconomic status are disproportionately affected by a higher prevalence of NCDs. A cross-country study involving 33 nations consistently demonstrates that individuals with lower socioeconomic status have increased odds of experiencing all seven morbidity outcomes in high-income countries, while some associations were less pronounced in the lower-middle-income countries (12). Additionally, research conducted in the United States (13) and Scotland (14) indicates that minority groups are at a significantly greater risks of developing chronic conditions compared to other populations, despite relatively low healthcare service utilization. These disparities are primarily attributed to differences in socioeconomic context.

As a populous developing country, China is confronted with an increasingly prominent challenge of population aging, necessitating urgent efforts in the prevention and management of NCDs (15). Since the 1990s, the prevalence of NCDs in China has steadily increased, with NCD-related mortality rising from 5.9 million in 1990 to 7.9 million in 2017 (16). This trend is particularly pronounced among adults aged above 45 years (12). In 2018, the prevalence of multiple chronic conditions among Chinese adults aged ≥45 was approximately 55.77% (17), while it reached around 65.16% among those aged ≥60 years old (18). Meanwhile, China is faced with a rapid surge in NCDs accompanied by notable regional and demographic disparities. The growth rate of chronic condition prevalence among rural residents surpasses that of urban counterparts, with rural areas also exhibiting higher rates of multimorbidity (19). However, treatment and control rates of NCDs in rural areas remain comparatively low (20). Studies indicate an alarming prevalence of NCDs in underdeveloped regions like Yunnan and Ningxia provinces, where certain NCDs occur at significantly higher rates than in the overall population (21, 22). Nevertheless, limited access to advanced medical equipment and inadequate availability of diverse medications contribute to low healthcare service utilization in these regions (23). Moreover, middle-aged and older adults from ethnic minority backgrounds in China demonstrate higher prevalence rates of NCDs and multimorbidity compared to the general population, with NCDs being a leading cause of mortality among several ethnic minority groups (24–26). Additionally, individuals from ethnic minority groups commonly encounter delays in seeking medical treatment or experience over-treatment due to misconceptions about NCDs (27).

Although current studies indicate significant disparities in the prevalence of NCDs, limitations in activities of daily living, and self-rated health among racial and ethnic older adults (24, 28), there remains a dearth of evidence regarding the impact of chronic conditions on ethnic minority population in later life. Furthermore, most of these studies have been conducted within high-income countries or urban settings, resulting in an inadequate understanding of the relationship between chronic conditions and health status within resource-limited environments. Given the escalating incidence and mortality rates associated with NCDs among ethnic minority population in China, it is imperative to investigate ethnic disparities in chronic conditions that may impact health outcomes in older age.

Yunnan Province, situated on the southwestern border of China, represents a prototypical region characterized by multi-ethnic settlement and economic underdevelopment. Within its 25 ethnic minority groups alongside the *Han* majority, 11 ethnic minority groups (*Wa, Pumi, Achang, Lisu, Lahu, Bulang, Jingpo, Nu, Jino, De’ang*, *and Drung* people) have undergone a remarkable developmental leap during the establishment of the People’s Republic of China in the 1950s. Transitioning directly from primitive or slave societies to socialist society has been their trajectory (29, 30). Primarily residing in remote mountainous areas with limited transportation and infrastructure, these ethnic groups face sluggish social development and persistent underdevelopment in both economic and social domains. Consequently, they have been longstanding targets for poverty alleviation efforts in China (30), including health initiatives aimed at reducing NCDs through strengthened prevention and control measures, alongside the provision of essential public health services for all (31).

Therefore, the investigation of both the prevalence of NCDs and their health impacts across diverse ethnic groups provides a robust foundation for enhancing policies aimed at mitigating health disparities and promoting equitable healthcare. Additionally, this research furnishes invaluable references for chronic disease prevention and control within similar context. The objectives of our study were to (1) examine the characteristics of chronic diseases and health status among adults aged ≥45, and (2) assess the impact of chronic conditions on the health status of different ethnic groups while controlling for sociodemographical factors.

## 2 Methods

### 2.1 Data and participants

A cross-sectional study was conducted in Yunnan Province, China, from July to December 2022, utilizing a structured questionnaire-based approach. The questionnaire consisted of three sections: demographic characteristics, chronic conditions, and health status. To ensure a representative sample, a stratified cluster sampling method was employed. Initially, five counties were randomly chosen in Yunnan Province based on their economic status, geographical features, and distribution of ethnic minority groups. From each selected county, two administrative villages were randomly chosen. The survey enrolled all residents aged ≥45 who had maintained local residency for at least 6 months. Individuals with cognitive or mental disorders, as well as those in a severe condition that prevented independent completion of all survey items, were excluded from the study.

The sample size was calculated using the formula: $Ν=\frac{\mu_{\alpha }^{2}\times Ρ(1-Ρ)}{\delta^{2}}\times \text{deff}$ , where *P* represents the prevalence rate of NCDs (50%) among individuals aged ≥45 in China for the year 2018 (32), and *δ* is set at 0.1 *P* = 0.05, *deff* = 3 and $\mu_{\alpha }$ =1.96. This calculation yielded a minimum required sample size of 1,382 subjects considering an anticipated non-response rate estimated at 20%.

Before data collection, investigators underwent rigorous training to ensure their proficiency in administering the process. Trained and experienced interviewers conducted face-to-face interviews with participants. A total of 2,710 participants were included in the final analysis after excluding 20 individuals with incomplete questionnaire responses, resulting in a validity rate of 99%.

### 2.2 Measures

#### 2.2.1 The number of NCDs

NCDs in this study are recognized by their non-transmissibility, prolonged duration, and gradual progression. To provide a more intuitive understanding of the impact of NCDs on individuals’ health, this study employed the number of NCDs as an indicator of chronic conditions. Data on participants’ NCD count were collected from a predefined list of five primary categories: cardiovascular diseases (hypertension, heart attack and dyslipidemia), cancers, chronic respiratory diseases (chronic obstructive pulmonary disease and asthma), diabetes, musculoskeletal disorders (rheumatoid arthritis, intervertebral disc disease, osteoporosis and gout) (33, 34). Additionally, an “other” option was included to capture any conditions self-reported by participants, provided that these conditions had been formally diagnosed by healthcare professionals (e.g., cerebrovascular disease, chronic kidney disease, chronic gastroenteritis, chronic anemia, hypothyroidism/hyperthyroidism, etc.) This allowed for generating a cumulative count of NCDs for each participant. Based on the definition of multimorbidity—defined as the presence of two or more NCDs in an individual (35)—the participants were categorized into four groups: 0, 1, 2, and 3 + NCDs.

#### 2.2.2 Ethnicity

Ethnicity in this study was determined through self-identification and categorized into *Han* majority group, *Zhiguo* ethnic minority group, and other ethnic minority group. Specifically, the study incorporated six major *Zhiguo* ethnic minority groups from the selected counties, namely *Wa, Lisu, Nu, Jino, Lahu*, and *Bulang* due to their similar historical development path and socioeconomic contexts. Additionally, individuals belonging to other ethnic minority groups such as *Akha*, *Zhuang*, *Dai*, *Yi* and *Hmong* were investigated based on the local ethnic composition of the selected counties.

#### 2.2.3 Health utility value

Health utility value, derived from the EuroQol five-dimension five-level (EQ-5D-5L) descriptive system, provides a comprehensive index for evaluating an individual’s health status (36). The EQ-5D-5L descriptive system is a self-reported scale consisting of five dimensions: Mobility, Self-Care, Usual Activities, Pain/Discomfort, and Anxiety/Depression. Each dimension is rated across five levels of severity, ranging from none to slight, moderate, severe, and extreme problems. By utilizing the Chinese value set for conversion purposes (37), measurements derived from this scale can be translated into health utility values, facilitating the assessment of an individual’s health status. Health utility values in China range from −0.391 to 1, with higher values reflecting better health status, where a score of 1 represents optimal health.

#### 2.2.4 Activities of daily living

Activities of Daily Living (ADL) refers to an individual’s ability to perform basic and routine daily activities and engage in routine life activities. It is commonly used as an indicator of functional status (38). In this study, ADL was assessed using the Activities of Daily Living Scale (ADLs) (39), a well-established tool for evaluating ADL among middle-aged and older individuals. The scale consists of two subscales: the Physical Self-Maintenance Scale (PSMS) and the Instrumental Activities of Daily Living Scale (IADL), which are efficient assessment instruments of functional abilities in older adults.

The PSMS includes six items related to personal care tasks, such as toileting, feeding, and dressing, while the IADL comprises eight items assessing more complex abilities, such as using telephone, shopping, and preparing food. Participants rated their level of self-care ability for each item on a scale ranging from “Independent” to “Completely Dependent,” with corresponding scores assigned from 1 to 4. The total ADL score was calculated by summing up the scores across all 14 items, resulting in a total score ranging from 14 to 56 where higher scores indicate lower levels of functional ability.

#### 2.2.5 Chronic pain

Chronic pain is characterized by its persistence beyond the normal healing period, lasting for more than 3 months (40). In this study, the intensity of chronic pain was assessed using the 10-point Numerical Rating Scale (NRS), a validated tool that quantifies pain on a range from 0 to 10, with higher values indicating greater severity (41). Regarding intensity classification, a score of 0 indicates no pain, while scores ranging from 1 to 3 indicate mild pain, scores from 4 to 6 denote moderate pain, and scores from 7 to 10 represent severe pain. Participants who reported experiencing chronic pain were asked to rate their current pain intensity using the NRS during the survey.

#### 2.2.6 Frailty

While the concept of frailty is now widely recognized, it remains a dynamic construct that perpetuates ongoing debates regarding its definition and characterization (42). In brief, frailty is a geriatric condition characterized by reduced physiological, functional and cognitive reserves, thereby increasing vulnerability to health stressors (43). The Tilburg Frailty Indicator (TFI) is a self-administered questionnaire that assesses multidimensional frailty in older adults across three dimensions: physical frailty (8 items), psychological frailty (4 items), and social frailty (3 items) (44). Each item is scored dichotomously as either “yes” (1 point) or “no” (0 points). The total frailty score ranges from 0 to 15, with higher scores indicating greater severity of frailty.

#### 2.2.7 Other variables

The study also examined several other variables, including age, gender, marital status, level of education, occupation, monthly household income and health literacy. Age was categorized into three groups: 45–59 years old, 60–74 years old, and ≥75 years old. Gender was grouped as male and female. Marital status was dichotomized into married and unmarried. Level of education was addressed as a binary variable: primary school or below versus junior high school or above. Occupation was classified as self-employed, worker, farmer, retired, unemployed and others. Monthly household income was divided into three categories: <3,000, 3,000–6,999, ≥7,000 RMB. Health literacy was assessed using the Brief Health Literacy Screen (BHLS), which is a verbally administered self-report measure of health literacy (45). The BHLS comprises three items rated on a 5-point Likert scale, except for the second item, which requires score reversal. The total score ranges from 3 to 15, with higher scores indicating greater levels of health literacy.

### 2.3 Statistical analysis

Data entry and validation checks were performed using EpiData 3.2 and data analysis was conducted using Stata 15.1. The distribution of demographic characteristics and health status indicators by the number of NCDs was described using counts with percentages and mean value ± standard deviation (SD), respectively. Chi-square tests were utilized to assess differences in the prevalence of NCDs across various demographic groups while the Kruskal-Wallis H test was used to examine differences in health status indicators among groups with differing numbers of NCDs within ethnic groups. In the final analysis, multivariable linear regression was used to assess the impact of NCDs, treated as a continuous variable, on three health status indicators: ADL, chronic pain, and frailty. Additionally, Tobit regression was employed to explore how the number of NCDs affects health utility value across diverse ethnic groups, while adjusting for covariates including age, gender, marital status, level of education, occupation, monthly household income, and health literacy. Statistical significance was determined at a significance level of *α* = 0.05.

## 3 Results

### 3.1 Demographic characteristics of the participants by the number of NCDs

Table 1 presents the demographic characteristics of the study participants. Among the total of 2,710 participants, their ages ranged from 45 to 99 years, with a mean age of 62.04 ± 10.22 years and a male-to-female ratio of 1:1.22. The study comprised individuals from various ethnic minority group, with participants from *Zhiguo* ethnic minority accounting for 38.23%, surpassing those from the *Han* majority (30.04%) and other ethnic minority groups (31.73%). A majority of the participants were married (75.76%). A significant proportion had only completed primary school or lower (79.40%), identified as farmers (70.17%), and reported a monthly household income below 3,000 RMB (54.26%). Notably, approximately 77% of participants demonstrated low levels of health literacy.

**Table 1:** Demographic characteristics of study participants by the number of NCDs.

Characteristic	Total	The number of NCDs				χ^2^	*p*-value
		0 disease	1 disease	2 diseases	3 + diseases		
Number of participants	2,710 (100.00)	1,141 (42.10)	858 (31.66)	434 (16.01)	277 (10.22)		
Age						170.053	< 0.001
45–59	1,161 (42.84)	641 (55.21)	328 (28.25)	123 (10.59)	69 (5.94)		
60–74	1,212 (44.72)	405 (33.42)	413 (34.08)	242 (19.97)	152 (12.54)		
≥ 75	337 (12.44)	95 (28.19)	117 (34.72)	69 (20.47)	56 (16.62)		
Gender						14.263	0.003
Male	1,218 (44.94)	552 (45.32)	375 (30.79)	190 (15.60)	101 (8.29)		
Female	1,492 (55.06)	589 (39.48)	483 (32.37)	244 (16.35)	176 (11.80)		
Ethnicity						75.378	< 0.001
*Han* majority	814 (30.04)	278 (34.15)	257 (31.57)	151 (18.55)	128 (15.72)		
Other ethnic minority	1,036 (38.23)	443 (42.76)	316 (30.50)	180 (17.37)	97 (9.36)		
*Zhiguo* ethnic minority	860 (31.73)	420 (48.84)	285 (33.14)	103 (11.98)	52 (6.05)		
Marital status						19.844	< 0.001
Married	2053 (75.76)	907 (44.18)	644 (31.37)	311 (15.15)	191 (9.30)		
Not married	657 (24.24)	234 (35.62)	214 (32.57)	123 (18.72)	86 (13.09)		
Level of education						27.404	< 0.001
Primary school or below	2,151 (79.40)	852 (39.61)	706 (32.82)	367 (17.06)	226 (10.51)		
Junior high school or above	558 (20.60)	288 (51.61)	152 (27.24)	67 (12.01)	51 (9.14)		
Occupation						90.237	< 0.001
Technician/office workers/civil servant	53 (1.96)	30 (56.60)	15 (28.30)	6 (11.32)	2 (3.77)		
Self-employed	86 (3.17)	45 (52.33)	21 (24.42)	11 (12.79)	9 (10.47)		
Worker	131 (4.84)	61 (46.56)	45 (34.35)	14 (10.69)	11 (8.40)		
Farmer	1901 (70.17)	844 (44.40)	603 (31.72)	296 (15.57)	158 (8.31)		
Retired	208 (7.68)	57 (27.40)	67 (32.21)	46 (22.12)	38 (18.27)		
Unemployed	297 (10.96)	87 (29.29)	95 (31.99)	59 (19.87)	56 (18.86)		
others	33 (1.22)	16 (48.48)	12 (36.36)	2 (6.06)	3 (9.09)		
Monthly household income (RMB)						7.595	0.269
< 3,000	1,470 (54.26)	615 (41.84)	461 (31.36)	246 (16.73)	148 (10.07)		
3,000–6,999	1,036 (38.24)	427 (41.22)	337 (32.53)	156 (15.06)	116 (11.20)		
≥ 7,000	203 (7.49)	98 (48.28)	60 (29.56)	32 (15.76)	13 (6.40)		
Health literacy						12.610	0.006
Low	2078 (76.68)	840 (40.42)	685 (32.96)	344 (16.55)	209 (10.06)		
Adequate	632 (23.32)	301 (47.63)	173 (27.37)	90 (14.24)	68 (10.76)		

The demographic characteristics of the study population, categorized by the number of NCDs, are also presented in Table 1. Among all participants, approximately 57.90% exhibited at least one NCD, while 16.01% were diagnosed with two NCDs, and 10.22% were affected by three or more NCDs. The prevalence of NCDs among the participants, in descending order, was as follows: hypertension (28.86%), intervertebral disc disease (14.32%), rheumatoid arthritis (14.06%), diabetes (7.82%), chronic gastroenteritis (6.05%), heart attack (4.87%), gout (3.73%), dyslipidemia (3.14%), cerebrovascular disease (2.58%), chronic obstructive pulmonary disease (2.21%), osteoporosis (1.81%), chronic anemia (0.70%), cancers (0.63%), chronic kidney disease (0.59%), hypothyroidism/hyperthyroidism (0.59%), and asthma (0.30%). It was observed that the number of NCDs increased significantly with advancing age (*p* < 0.001). In terms of ethnicity, individuals from the *Han* majority showed a larger number of NCDs compared to those from the *Zhiguo* ethnic minority and other ethnic minority groups (*p* < 0.001). Furthermore, a higher prevalence of NCDs was found among females, unmarried individuals and those with lower levels of health literary (*p* < 0.01). Conversely, individuals employed as technicians, office workers, civil servants and self-employed demonstrated a relatively lower number of NCDs (*p* < 0.001).

### 3.2 Ethnic disparities in health status by the number of NCDs

Table 2 illustrates the distribution of health status across different ethnic groups based on the number of NCDs. In the overall surveyed participants, the mean values for health utility (0.94, SD = 0.11), ADL (15.86, SD = 4.25), chronic pain (2.02, SD = 2.18) and frailty (3.06, SD = 2.50) were reported, respectively. Significant variations in health status regarding health utility value, ADL, chronic pain, and frailty were observed across groups stratified by the number of NCDs (*p* < 0.001). Health utility value demonstrated a decline with an increasing number of NCDs, while scores for ADL, chronic pain, and frailty exhibited an increase with a higher accumulation of NCDs.

**Table 2:** Ethnic disparities in health status by the number of NCDs.

Ethnicity	Health status	Total	The number of chronic diseases				*H*	*P*-value
			0 disease	1 disease	2 diseases	3 + diseases		
All participants	Health utility value	0.94 ± 0.11	0.97 ± 0.07	0.95 ± 0.09	0.91 ± 0.15	0.89 ± 0.016	289.641	< 0.001
	ADL	15.86 ± 4.25	15.46 ± 3.58	15.72 ± 3.71	16.62 ± 5.27	16.79 ± 5.94	34.916	< 0.001
	Chronic pain	2.02 ± 2.18	1.27 ± 1.89	2.16 ± 2.10	2.84 ± 2.27	3.40 ± 2.18	332.117	< 0.001
	Frailty	3.06 ± 2.50	2.37 ± 2.00	3.15 ± 2.45	3.79 ± 2.75	4.51 ± 3.05	202.930	< 0.001
*Han* majority	Health utility value	0.94 ± 0.11	0.96 ± 0.08	0.94 ± 0.09	0.91 ± 0.13	0.89 ± 0.15	71.123	< 0.001
	ADL	15.78 ± 4.50	15.44 ± 3.44	15.76 ± 4.54	15.74 ± 4.25	16.60 ± 6.32	2.709	0.439
	Chronic pain	2.10 ± 2.21	1.37 ± 1.92	1.93 ± 2.04	2.70 ± 2.35	3.30 ± 2.30	79.388	< 0.001
	Frailty	3.19 ± 2.59	2.42 ± 2.14	3.37 ± 2.62	3.52 ± 2.56	4.13 ± 3.00	45.558	< 0.001
*Zhiguo* ethnic minority	Health utility value	0.94 ± 0.12	0.97 ± 0.07	0.94 ± 0.10	0.90 ± 0.17	0.89 ± 0.16	94.932	< 0.001
	ADL	15.92 ± 3.80	15.21 ± 2.71	15.73 ± 3.05	17.39 ± 5.64	17.06 ± 4.90	60.048	< 0.001
	Chronic Pain	2.27 ± 2.28	1.51 ± 2.09	2.58 ± 2.23	2.82 ± 2.25	3.69 ± 2.19	115.386	< 0.001
	Frailty	3.37 ± 2.61	2.65 ± 2.19	3.40 ± 2.45	4.22 ± 2.96	4.96 ± 3.07	85.026	< 0.001
Other ethnic minority	Health utility value	0.96 ± 0.10	0.98 ± 0.06	0.95 ± 0.09	0.91 ± 0.14	0.88 ± 0.18	117.042	< 0.001
	ADL	15.88 ± 4.51	15.73 ± 4.36	15.68 ± 3.56	16.55 ± 5.78	16.77 ± 6.78	2.712	0.438
	Chronic pain	1.64 ± 1.96	0.94 ± 1.57	1.90 ± 1.94	3.07 ± 2.17	3.12 ± 1.82	141.588	< 0.001
	Frailty	2.57 ± 2.17	2.05 ± 1.63	2.67 ± 2.22	3.42 ± 2.56	4.60 ± 3.06	64.364	< 0.001

In term of ethnicity, the distribution of health status revealed that the mean health utility value for participants from other ethnic minority groups was 0.96 (SD = 0.10), exceeding that of both the *Han* majority and *Zhiguo* ethnic minority groups. Additionally, individuals from the *Zhiguo* ethnic minority groups had higher mean scores for ADL (15.92, SD = 3.80), chronic pain (2.27, SD = 2.28), and frailty (3.37, SD = 2.61) compared to those from both the *Han* majority and other ethnic minority groups. Regarding health status distinctions based on the number of NCDs, no statistically significant differences in ADL scores were detected both the *Han* majority and other ethnic minority groups; however, all other health statuses exhibited significant differences consistent with the overall population trends (*p* < 0.001).

### 3.3 Ethnic disparities in the impact of the number of NCDs on health status

**Figure 1:**
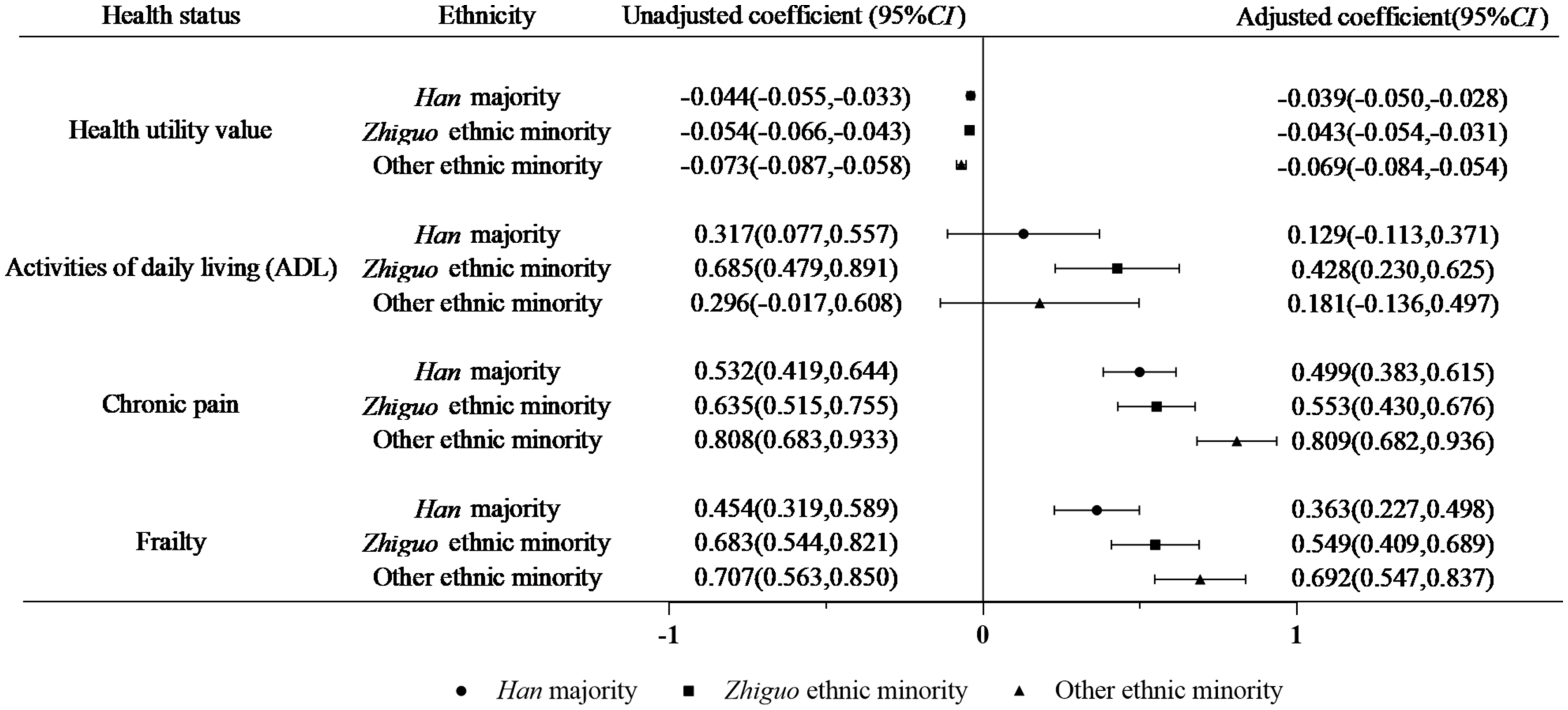
Impact of the number of NCDs on health status by ethnic groups (adjusted for age, gender, marital status, level of education, occupation, monthly household income and health literacy).

Ethnic disparities in the impact of the number of NCDs on health status are illustrated in Figure 1. A significantly negative association was observed between the number of NCDs and the health utility value across all ethnic groups: the *Han* population (*β* = −0.039, *p* < 0.001), *Zhiguo* ethnic minority (*β* = −0.043, *p* < 0.001), and other ethnic minority groups (*β* = −0.069, *p* < 0.001). In contrast, a positive correlation was identified solely within the *Zhiguo* ethnic minority groups between the number of NCDs and ADL scores (*β* = 0.428, *p* < 0.001). Meanwhile, there was a positive association between the number of NCDs and chronic pain score among all three populations: *Han* majority (*β* = −0.499, *p* < 0.001), *Zhiguo* ethnic minority (*β* = 0.553, *p* < 0.001), and other ethnic minority groups (*β* = 0.809, *p* < 0.001). Similarly, frailty scores exhibited a positive relationship with the number of NCDs for all three populations: *Han* majority (*β* = 0.363, *p* < 0.001), *Zhiguo* ethnic minority (*β* = 0.549, *p* < 0.001), and other ethnic minority groups (*β* = 0.692, *p* < 0.001).

## 4 Discussion

This study aimed to investigate the prevalence of NCDs among diverse ethnic groups and their impact on health status in underdeveloped regions of China. Participants were categorized into three distinct ethnic groups: the *Han* majority, the *Zhiguo* ethnic minority, and other ethnic minority. The findings not only provide a comprehensive assessment of chronic disease prevalence and multimorbidity rates within these groups but also elucidate the influence of chronic diseases on health status across four dimensions: health utility value, ADL, chronic pain, and frailty.

Overall, the prevalence of NCDs among adults aged ≥45 in this study was 57.90%, notably higher than the national average prevalence among the corresponding population in China (44.46%) from 2018 (46). This difference indicates a substantial burden of NCDs in underdeveloped areas and highlighting the need for enhanced prevention and management strategies tailored for these conditions. In this population, aside from hypertension, the profile of NCDs is predominantly characterized by musculoskeletal disorders, particularly intervertebral disc diseases and rheumatoid arthritis. The pattern markedly differs from the national prevalence of NCDs among the corresponding population in China, where dyslipidemia (14.7%) ranks as the second most prevalent condition after hypertension (22.5%) (32). The observed discrepancy in disease prevalence may be attributable to the study population’s substantial engagement in physically demanding agricultural and animal husbandry activities, which are likely to contribute to the elevated incidence of musculoskeletal disorders (29, 47).

However, the prevalence of multimorbidity among adults aged ≥45 was 26.23%, which is considerably lower than the rates reported in recent studies by Liu et al. (48), Cui et al. (49) and Wang et al. (17), which reported multimorbidity rates of 63, 55.8, and 55.77%, respectively. The disparity in multimorbidity findings may be attributed to the fact that participants in this study primarily resided in ethnic minority areas within Yunnan province. Notably, individuals belonging to the *Zhiguo* ethnic minority group and other ethnic minority group exhibited a lower number of NCDs compared to the *Han* population. This suggests that *Han* residents within the same region might face additional risk factors for NCDs warranting further investigation into potential biological, behavioral or environmental determinants specific to this population subgroup. Meanwhile, it is crucial to acknowledge that the lower prevalence of NCDs observed among ethnic minority groups, particularly the *Zhiguo* ethnic minority group, may potentially be influenced by their comparatively lower levels of health literacy as well as limited access to healthcare services prevalent in these regions. These factors may contribute to the under-detection of multimorbidity (50), highlighting the need for targeted health education and improved healthcare accessibility in these communities.

The prevalence rates of NCDs and multimorbidity in females observed in the current study (60.52 and 28.15%, respectively) were significantly higher than those in males (54.68 and 23.89%, respectively). These findings align with previous studies conducted in China (48, 51). This gender disparity may be attributed to the physiological changes experienced by women during perimenopause, characterized by decreased estrogen levels (52). Additionally, women’s higher utilization of preventive health services, such as physical examinations, likely contributes to the increased detection rate of multimorbidity compared to men (53).

The study also revealed that unmarried groups exhibited a higher prevalence of NCDs and multimorbidity (64.38 and 31.81%, respectively) compared to the married group (55.82 and 24.45%, respectively). The discrepancy may be explained by the lack of familial support and psychological solace among unmarried middle-aged and older adults, as well as their heightened engagement in health-compromising behaviors. Moreover, unmarried individuals often face greater socioeconomic barriers, limiting their access to healthcare services and hindering their ability to make health-promoting choices (54, 55).

To further assess the health status of Chinese middle-aged and older adults with and without NCDs, this study compared health utility, ADL, chronic pain, and frailty among the *Han* majority, *Zhiguo* ethnic minority, and other ethnic minority groups. Overall, individuals from six *Zhiguo* ethnic groups reported higher level of ADL limitations, chronic pain, and frailty. Previous research has indicated that the life expectancy of *Zhiguo* ethnic groups is lower than that of the general Chinese population, potentially suggesting a higher risk of developing health problems, including NCDs at a younger age within these ethnic minority population (26, 56). In addition, this study found a statistically significant positive correlation between the number of chronic diseases and ADL scores specifically among *Zhiguo* ethnic minority groups. Most *Zhiguo* ethnic minority groups reside in remote areas characterized by limited access to educational and healthcare resources. This geographical and infrastructural context often results in restricted health literacy and self-care capabilities among these populations, rendering them more vulnerable to disease-related risks (29, 30). Consequently, individuals from these communities exhibit a heightened reliance on external support for the management of chronic conditions, such as adherence to prescribed medication regiments.

Delayed or inadequate diagnosis and treatment of chronic diseases further contribute to rapid disease progression and preventable disability, particularly within these vulnerable populations (57, 58). Therefore, it is imperative to prioritize the healthcare needs of *Zhiguo* ethnic groups by implementing early-age screening and management programs targeting chronic diseases. Such interventions could mitigate the progression of disease and reduce the burden of disability within this undeserved population.

The results of this study also indicated that, after controlling for confounding factors, the impact of multiple chronic diseases on the health status of ethnic minority groups—measured in terms of health utility, chronic pain, and frailty—was found to be more pronounced. Although the *Han* majority reporting the highest prevalence of multimorbidity, their health status was the least affected by the number of chronic diseases. The observed difference could be linked to the *Han* majority’s better accessibility and higher utilization of healthcare services for chronic diseases due to advantages in transportation and language (59, 60), which likely improve chronic disease management, alleviate pain, prevent frailty, and enhance quality of life. In contrast, although the prevalence of multimorbidity were lower among both *Zhiguo* and other ethnic minority groups, these populations experienced a greater impact on health utility, ADL, chronic pain, and frailty compared to the *Han* majority. This suggests that the chronic diseases suffered by ethnic minority population are more severe and have a greater effect on their overall health status. It is also possible that limited health awareness and under-diagnosis of chronic diseases among ethnic minority population (61, 62) may account for the observed findings, with individuals experiencing reduced ADL, chronic pain, and frailty even before diagnosis or treatment, thereby mitigating the impact of chronic diseases on health outcomes. Moreover, the *Zhiguo* ethnic minority reported a higher prevalence of physical disabilities compared to other groups, despite lower self-reported rates of pain and frailty. This may be linked to historical and cultural factors, where harsh living conditions and cultural norms among the *Zhiguo* people foster a tendency to endure suffering without outwardly expressing subjective feelings (63). These findings underscore the importance of addressing the unique healthcare needs of this population and ensuring equitable access to care.

From an ethnic disparity perspective, this study reveals that, although the *Han* majority has a higher prevalence of chronic diseases, the negative health effects are more severe among ethnic minority groups. This highlights existing health inequities among different ethnic groups, emphasizing targeted interventions to address these divergences. The implications of these findings can be extrapolated to similar contexts where diverse ethnic minority populations face resource-constrained settings.

However, several limitations must be acknowledged. Firstly, further investigations are recommended to mitigate potential selection bias among the remaining 25 ethnic minority groups in Yunnan Province. Secondly, the reliance on self-reported data may prompt potential biases such as social desirability and recall distortion. Finally, the assessment of participants’ chronic conditions is solely relied on the count of NCDs, without considering the differential impact, severity or duration associated with specific disease types. Future research should consider incorporating these factors, such as the types of NCDs, their coexistence patterns, and the duration of each disease, to provide a more comprehensive and nuanced understanding of the impact of chronic conditions on individuals’ health.

## 5 Conclusion

In summary, the prevalence of NCDs among adults aged ≥45 in Yunnan province, an economically underdeveloped region in China, is notably higher. Significant ethnic disparities are evident, particularly in the relationship between the number of chronic conditions and their negative health impacts. Individuals from the *Zhiguo* ethnic minority experience worse health outcomes—such as reduced health utility, ADL, chronic pain, and frailty—compared to the *Han* majority. These findings underscore the urgent need for tailored action and intensified efforts to address health inequalities in multi-ethnic regions by enhancing multimorbidity prevention and management strategies.

## Data Availability

The raw data supporting the conclusions of this article will be made available by the authors, without undue reservation.
